# Solid Pseudopapillary Tumor of the Pancreas in a 25-Year-Old Female: A Rare Entity of Pancreatic Tumors

**DOI:** 10.7759/cureus.14747

**Published:** 2021-04-29

**Authors:** Dimitrios Massaras, Zoi Masourou, Maria Papazian, Grigorios Psarras, Andreas Polydorou

**Affiliations:** 1 Surgery, Aretaieio University Hospital, National and Kapodistrian University of Athens School of Medicine, Athens, GRC; 2 Anesthesiology, Aretaieio University Hospital, National and Kapodistrian University of Athens School of Medicine, Athens, GRC; 3 Pathology, Aretaieio University Hospital, National and Kapodistrian University of Athens School of Medicine, Athens, GRC; 4 1st Department of Radiology, Aretaieio University Hospital, National and Kapodistrian University of Athens School of Medicine, Athens, GRC

**Keywords:** solid pseudopapillary neoplasm, whipple, β-catenin, pancreatic tumor, cystic neoplasms

## Abstract

Solid pseudopapillary neoplasms (SPMs) of the pancreas are extremely rare tumors of the pancreas that typically affect young women and have a favorable prognosis. Herein, we report a 25-year-old female with solid pseudopapillary tumor of the pancreas who presented with atypical epigastric pain. The patient underwent pancreatoduodenectomy (Whipple procedure). She remained asymptomatic and showed no signs of disease after one year of follow-up. This type of pancreatic tumors is amenable to cure after complete surgical resection, even in cases with capsular invasion, unlike any other malignant tumors of the pancreas.

## Introduction

Solid pseudopapillary neoplasm (SPN) of the pancreas is a rare tumor of the pancreas with low malignant potential that typically affects young women in their third decade of life. They have a low malignant potential and usually are asymptomatic or present with minimal atypical symptoms [1]. In 1996, the World Health Organization classified those tumors as borderline neoplasms of the exocrine pancreas with respect to their metastatic and recurrence potential [2]. SPNs account for 4% of all cystic pancreatic lesions, and they are most commonly located in the body or tail of the pancreas [3,4]. Τhe radiological diagnosis of these tumors can be very challenging. The typical image in computed tomography (CT) or magnetic resonance (MR) is a mass that consists of both solid and cystic elements surrounded by a well-defined capsule. These neoplasms can easily be misdiagnosed as adenocarcinoma of the pancreas [5]. Ιn contrast to other pancreatic cysts in which only surveillance is recommended, in SPNs, surgery is recommended as the optimal management [6].

## Case presentation

**Figure 1:**
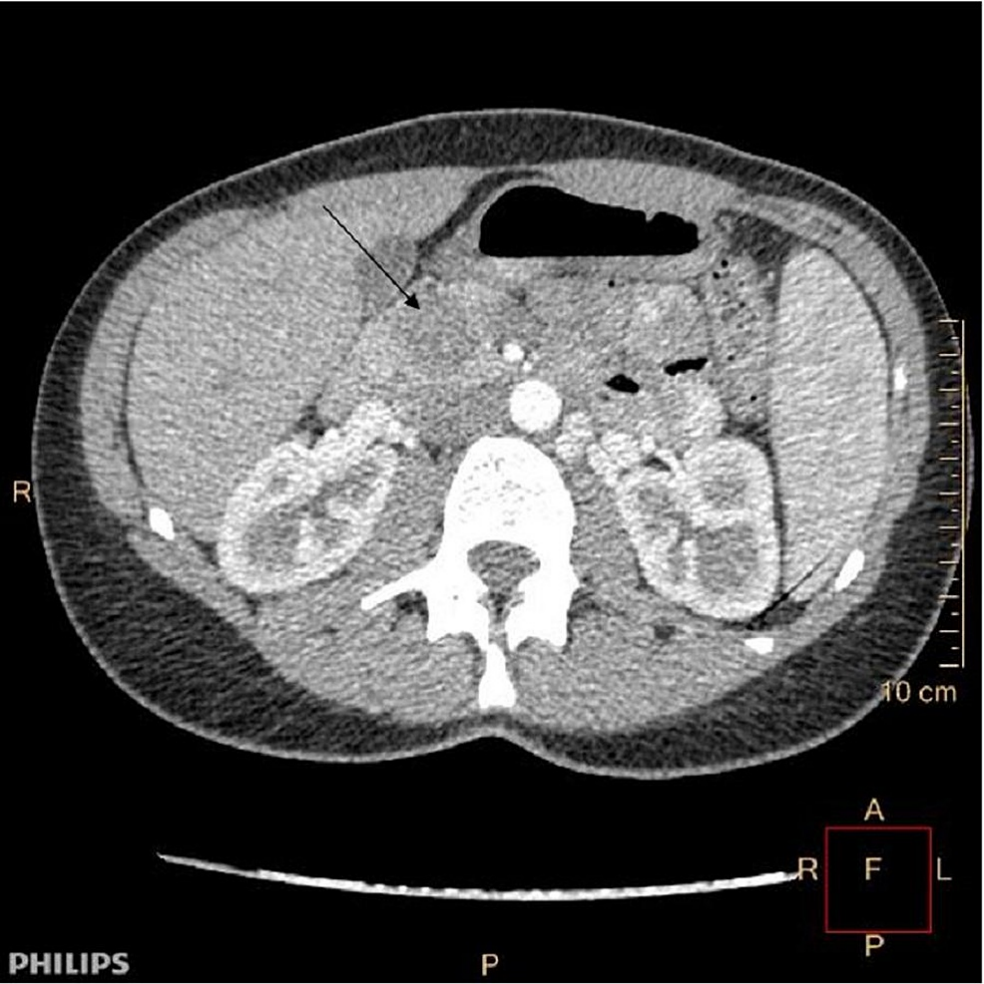
CT scan arrow pointing the pancreatic tumor in the head of the pancreas

**Figure 2:**
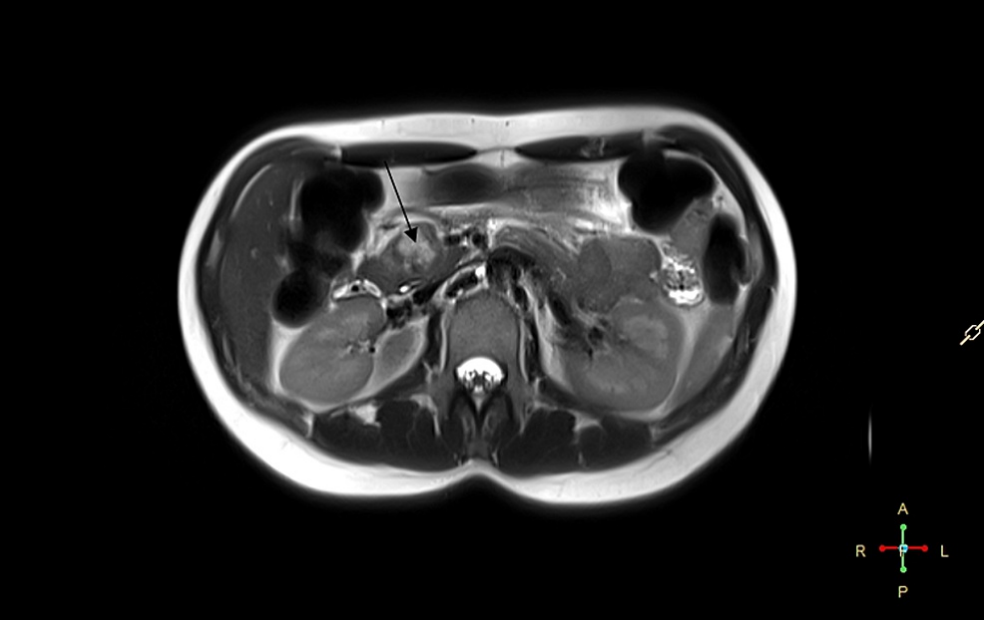
MRI scan arrow pointing the well-described pancreatic tumor

A 25-year-old female with no prior medical history presented with atypical complaints of epigastric pain. The pain was constant and periodic in nature. There was no laboratory abnormality, and there was no family history of cancer. The patient was afebrile, and there was no complaint of vomit, dizziness, and gastrointestinal upset. On physical examination, there was no palpable mass in the abdominal cavity and no evidence of any tenderness. General examination was unremarkable. The ultrasound initially discovered a heterogeneous mass in the pancreatic head that could not be characterized. A computed tomography scan was then performed that described a mass in the head of the pancreas measuring 2.5 cm (Figure 1). Also, a magnetic resonance scan was performed that described a mass in the head of the pancreas measuring 2.8 cm x 2.8 cm x 2.5 cm with well-defined borders and mild contrast enhancement (Figure 2).

**Figure 3:**
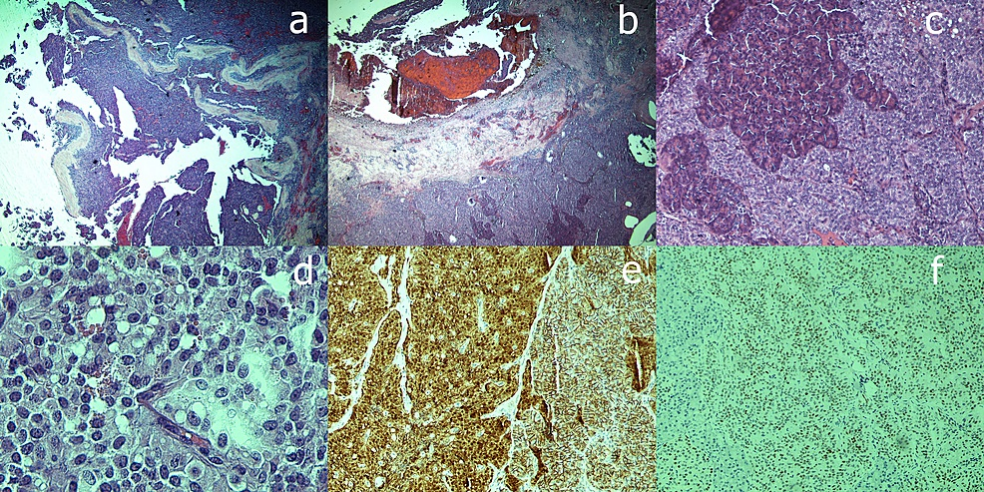
(a) Detached neoplastic cells forming pseudopapillae and protruding into pseudocystic spaces - hematoxylin-eosin staining with magnification x40. (b) Eosinophilic cells of the tumor surrounding fibrovascular cords - hematoxylin-eosin staining with magnification x40. (c) Tumor-infiltrating pancreatic parenchyma - hematoxylin-eosin staining with magnification x200. (d) Intracytoplasmic PAS-D(+) hyaline globules - hematoxylin-eosin staining with magnification x400. (e) Neoplastic cells with nuclear/cytoplasmic expression of β-catenin - immunohistochemical staining with magnification x200. (f) Weak nuclear positivity for PgR - immunohistochemical staining with magnification x200. PAS-D, Periodic acid-Schiff-diastase; PgR, progesterone receptor.

An endoscopic ultrasound was then performed as there was no definite diagnosis of the tumor, and fine-needle aspiration was performed that characterized the tumor as a solid pseudopapillary tumor of the pancreas. In the multidisciplinary team (MDT) meeting, a surgical resection was decided, and the patient underwent Whipple's procedure. On dissection of the pancreatic head, a solitary tan-white well-circumscribed tumor measuring 2.8 cm x 2 cm x 1.4 cm was revealed. The tumor was mainly solid containing small cystic partly hemorrhagic spaces. Microscopically, the neoplasm consisted of solid nests of poorly cohesive monomorphic eosinophilic cells with low nuclear atypia surrounding hyalinized fibrovascular cords (Figures 3a, 3b). The cells’ nuclei were round to ovoid with finely dispersed chromatin and inconspicuous nucleoli. Intracytoplasmic PAS-D(+) (periodic acid-Schiff-diastase) hyaline globules were identified (Figure 3d). The mitotic activity was scarce. Detached neoplastic cells formed pseudopapillae that protruded into pseudocystic spaces (Figure 3a). Areas of intratumoral hemorrhage were accompanied by cholesterol crystals and foamy histiocytes. The tumor infiltrated the surrounding pancreatic parenchyma (Figure 3c). Perineural and vascular invasion was observed. On immunohistochemical analysis, the neoplastic cells showed nuclear/cytoplasmic expression of β-catenin (Figure 3e), weak nuclear positivity for progesterone receptor (PgR) (Figure 3f), and intense cytoplasmic expression of vimentin. A1-antitrypsin highlighted the cytoplasmic hyaline globules. Chromogranin, synaptophysin, and CD99 immunohistochemical stains were negative. CD117 nuclear expression was also considered negative.

In the otherwise unremarkable adjacent pancreatic parenchyma, a few low-grade pancreatic intraepithelial neoplasia (PanIN) foci of the peripheral pancreatic ducts were observed. Sixteen peripancreatic lymph nodes were negative. The postoperative course of the patient was uncomplicated and was discharged on the seventh postoperative day. After one year of follow-up, the patient has no sign of local recurrence or metastasis from the primary tumor. The patient was also tested genetically for any possible genetic association of this tumor to any known genetic syndrome. The molecular test was performed with analysis of DNA sequences. There were two focal changes in the DNA sequence that were recognized. The first focal change was in the expression of protein cyclin-dependent kinase inhibitor 2A (CDKN2A) that is only described in the international literature in 0.01% of the population in patients suffering from melanoma and acute lymphoblastic leukemia. However, the data to relate the patient with this focal change are not sufficient, so this finding could not be characterized. Additionally, the second focal change in the genome was described in the expression of neurofibromin 1 (NF1) protein in exon 49 that it is not expected to cause any abnormal expression in the function and structure of protein NF1. The findings of this genetic test were not conclusive, and no genetic background was recognized for the expression of this pancreatic neoplasm.

## Discussion

Solid pseudopapillary tumor of the pancreas is an extremely rare neoplasm of the pancreas. It represents approximately 1% of all the tumors of the pancreas. It was first described in 1959 by Frantz, and many different names were given to these neoplasms until 1996 that WHO categorized those neoplasms as solid pseudopapillary tumors of the pancreas [7]. The origin of these tumors is currently unknown. Many investigators favor the theory that SPNs originate from primordial cells, whereas others suggest an extrapancreatic origin [8]. The male-to-female ratio is 1:10, and the mean age at presentation is 23 years. Although most SPNs show benign behavior, malignancy can occur in about 15% of cases, manifesting as metastases or invasion of adjacent structures [8]. The most common metastatic sites are the liver and the omentum. Histologically, SPNs exhibit unique characteristics. They are positive for α1-antitrypsin, CD56, CD10, and vimentin [8]. Although SPNs have locally aggressive features, these tumors have a low-grade malignant potential and tend to have a favorable prognosis, even in the presence of metastatic disease. However, due to the rarity of SPNs and the overall excellent prognosis, reliable prognostic factors to predict malignant biological behavior remain undetermined. There is only one recent interesting and promising analysis that tried to identify miRNAs that are differentially expressed in metastatic tumors compared with localized tumors. This molecular characteristic could potentially serve as a molecular marker or signature used to identify tumors that are at increased risk to develop metastases, thereby requiring long-term surveillance [9]. Despite that, the optimal management of these tumors remains the surgical resection. Overall, five-year survival is as high as 97% in patients undergoing surgical resection even in the case of distant hepatic metastasis or local recurrence [10].

## Conclusions

SPNs should always be considered in the differential diagnoses of pancreatic tumors especially in young adults despite the rarity of these neoplasms. An MDT approach was required to direct and achieve appropriate management. Surgical resection of these tumors offers the best therapeutic result with the best prognosis. Finally, the genetic background of these tumors can be associated with known DNA mutations; therefore, genetic screening in young adults is advised.
